# Application of NICE or SNC guidelines may reduce the need for computerized tomographies in patients with mild traumatic brain injury: a retrospective chart review and theoretical application of five guidelines

**DOI:** 10.1186/s13049-019-0673-8

**Published:** 2019-11-04

**Authors:** Sebastian Svensson, Tomas Vedin, Linus Clausen, Per-Anders Larsson, Marcus Edelhamre

**Affiliations:** 1 Department of Clinical Sciences, Medical Faculty, Lund University, Lund, Sweden; 2 Medical Faculty, Lund University, Lund, Sweden

## Abstract

**Background:**

Traumatic brain injuries continue to be a significant cause of mortality and morbidity worldwide. Most traumatic brain injuries are classified as mild, with a low but not negligible risk of intracranial hemorrhage. To help physicians decide which patients might benefit from a computerized tomography (CT) of the head to rule out intracranial hemorrhage, several clinical decision rules have been developed and proven effective in reducing the amount of negative CTs, but they have not been compared against one another in the same cohort as to which one demonstrates the best performance.

**Methods:**

This study involved a retrospective review of the medical records of patients seeking care between January 1 and December 31, 2017 at Helsingborg Hospital, Sweden after head trauma. The Canadian CT Head Rule (CCHR), the New Orleans Criteria (NOC), the National Emergency X-Radiography Utilization Study II (NEXUS II), the National Institute of Health and Care Excellence (NICE) guideline and the Scandinavian Neurotrauma Committee (SNC) guideline were analyzed. A theoretical model for each guideline was constructed and applied to the cohort to yield a theoretical CT-rate for each guideline. Performance parameters were calculated and compared.

**Results:**

One thousand three hundred fifty-three patients were included; 825 (61%) CTs were performed, and 70 (5.2%) cases of intracranial hemorrhage were found. The CCHR and the NOC were applicable to a minority of the patients, while the NEXUS II, the NICE, and the SNC guidelines were applicable to the entire cohort. A theoretical application of the NICE and the SNC guidelines would have reduced the number of CT scans by 17 and 9% (*P* = < 0.0001), respectively, without missing patients with intracranial hemorrhages requiring neurosurgical intervention.

**Conclusion:**

A broad application of either NICE or the SNC guidelines could potentially reduce the number of CT scans in patients suffering from mTBI in a Scandinavian setting, while the other guidelines seemed to increase the CT frequency. The sensitivity for intracranial hemorrhage was lower than in previous studies for all guidelines, but no fatality or need for neurosurgical intervention was missed by any guideline when they were applicable.

## Background

Traumatic brain injury (TBI) is a common occurrence in emergency departments (EDs), resulting in an estimated 2.1 million hospital admissions (287.2/100,000) and 82,000 deaths (11.7/100,000) in Europe in 2012 [1]. TBI can be categorized as minimal, mild, moderate, or severe, depending on the patient’s level of consciousness according to the Glasgow Coma Scale (GCS) and the presence or the absence of certain characteristics. Minimal TBI is defined as GCS 15 without loss of consciousness (LOC) or amnesia. Mild TBI (mTBI) is defined as GCS 14–15, associated with amnesia, brief LOC, or impaired alertness or memory. Moderate TBI is defined as GCS 9–13, with an extended period of LOC or the presence of a neurological deficit. Severe (and critical) TBI is defined as GCS 3–8 [2].

Additional definitions of mTBI have been proposed, but consensus is lacking [3, 4]. Minimal TBI and mTBI constitute 71–97.5% of the cases, of which 4.8–8% will have an intracranial hematoma, and approximately 1% will require neurosurgical intervention [5–7]. The preferred diagnostic modality in the evaluation of TBI patients is computerized tomography (CT) of the head because it is readily available, requires less scanning time compared with magnetic resonance imaging, and shows excellent performance in diagnosing acute intracranial injuries [8]. The drawbacks of CT are its possible association with the development of neoplasms (because of exposure to ionizing radiation) and the higher costs for the healthcare system [9, 10].

To safely reduce the number of CT scans, various clinical guidelines have been developed. The most studied ones are the Canadian CT Head Rule (CCHR) and the New Orleans Criteria (NOC). Both originate from North America and show a significant reduction of CT scans in their derivation cohorts [6, 11]. Additional guidelines, such as those from the National Emergency X-Radiography Utilization Study II (NEXUS II), the National Institute for Health and Care Excellence (NICE), and the Scandinavian Neurotrauma Committee (SNC), have been developed in more recent years [12–14]. The SNC guideline is the first to include the biomarker S100B as an adjunct to clinical evaluation. This has shown potential reductions in the need for CT and in healthcare costs [15, 16].

These guidelines have been compared in different constellations and settings, especially the CCHR with the NOC. These comparisons have yielded slightly different results, but generally, the CCHR shows a higher specificity but similar sensitivity as the NOC [17–21]. The other mentioned guidelines have been less studied. They all exhibit perfect or near perfect sensitivity to injuries requiring neurosurgery but with a considerable variety in specificity, both among different guidelines and different studies on the same guideline [22–28].

The aim was to compare the performance and the number of CTs recommended by the NOC, the CCHR, NEXUS II, the NICE, and the SNC in a Scandinavian setting. The primary outcome measure was intracranial injury requiring neurosurgical intervention, and the secondary outcome measure was any intracranial hemorrhage shown on CT.

## Methods

**Fig. 1:**
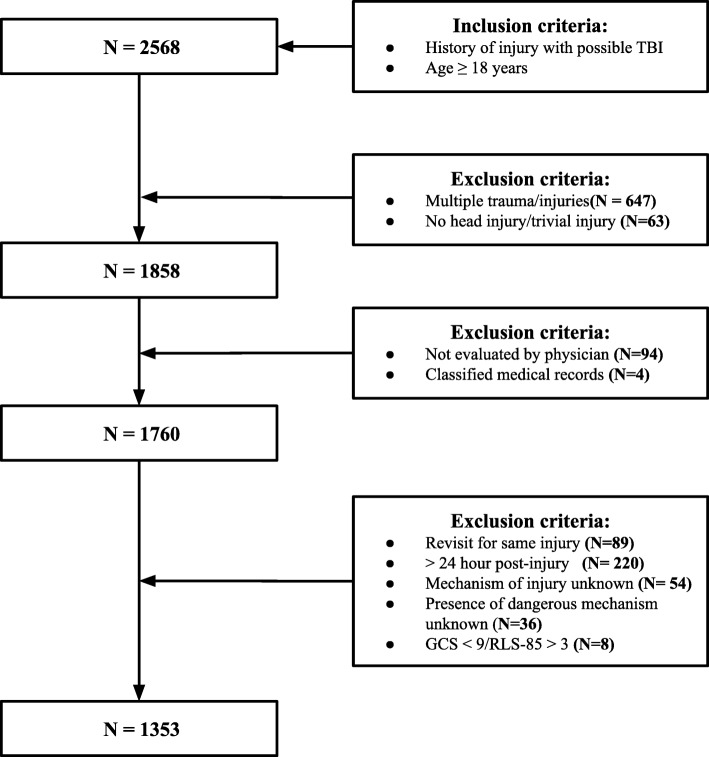
Inclusion and exclusion criteria. TBI = traumatic brain injury, GCS = Glasgow Coma Scale, RLS-85 = Reaction Level Scale 85

This study involved a retrospective review of the medical records of patients seeking care in the ED of Helsingborg Hospital, Sweden due to an isolated head injury sustained between January 1 and December 31, 2017. The hospital serves approximately 250,000 inhabitants, and its ED has approximately 60,000 visits each year. The hospital’s guideline for managing patients with minimal to moderate TBI is adapted from the most recent SNC guideline. Figure 1 shows the inclusion and the exclusion criteria.

In the first step, multiple trauma patients were excluded because this group is routinely managed according to the Advanced Trauma Life Support (ATLS). Patients with no history of a head injury or who suffered only trivial injuries (for example, superficial cuts) were also excluded in this step. In the second step, patients were excluded if they were only treated by a triage nurse, left at their own discretion without being examined by a physician, or their medical records were classified. In the third step, patients were excluded based on different factors that would make them ineligible for evaluation by at least one of the studied guidelines.

We chose which parameters to extract based on their presence in any of the guidelines that we set out to evaluate. Appendix 1 presents the full list of the parameters.

The missing data in any parameter was interpreted as the absence of pathological findings. This decision was made based on our experience that physicians in the ED are pragmatic when writing medical records and only report positive findings, not negating negative ones. The parameters chosen for extraction are routinely assessed and spontaneously reported when caring for TBI patients, and we deem it unlikely that these were systematically overlooked in the dataset.

A dangerous mechanism was based on the definition provided by Stiell et al. [6]. An abnormal level of alertness and abnormal behavior were defined as suggested by Mower et al. [12].

Intracranial hemorrhage was defined as its presence showing on a CT scan. The absence of intracranial hemorrhage meant either a negative CT scan upon the patient’s arrival or the omission of CT at the treating physician’s discretion. Neurosurgical intervention pertained to any surgical intervention or intubation performed because of intracranial injury. Death due to TBI was defined as death tribute to the head injury during the actual hospital admission.

The information was obtained from the records of the physicians managing the patients in the ED, together with the laboratory results and the radiology reports. Notes from up to 1 year prior to the visit was screened for medications and comorbidities when not found in current note. In case of admission, other relevant medical records were also reviewed, such as round notes and discharge summaries from admission until discharge, either from the ED or the ward, from the current stay. In cases of missing data in the records of the physicians managing the patients, the entries made by the medical personnel other than the treating physicians were reviewed to fill in the data gap.

To enable analysis and comparison of the guidelines, the following assumptions were made:

1. Since the Reaction Level Scale 85 (RLS-85) is preferred for determining a patients level of consciousness in the study hospital, the level of consciousness had to be converted to the GCS in the following manner [14, 29]:

- RLS 1 = GCS 15,
- RLS 2 = GCS 14, and
- RLS 3 = GCS 9–13.

2) The level of consciousness was retrieved once by a physician, approximately 10–60 min after arrival at the ED. Where level of consciousness could not be interpreted from the physicians note, an estimate done by the triage nurse upon arrival was used.
3) Treatment with warfarin was extended to include treatment with new oral anticoagulants (NOACs) and any dose of low molecular-weight heparin.
4) Any amnesia was considered a deficit in short-term memory when evaluating the NOC.
5) Indirect signs of post-traumatic seizure (involuntary voiding, postictal state, or biting of the tongue) were considered post-traumatic seizure.
6) Any kind of amnesia whose duration exceeded 30 min was included as a risk factor for the CCHR and the NICE guideline.
7) Patients for whom the analysis of S100B was recommended by the SNC guideline, but no actual analysis was done was interpreted as qualifying for CT.
8) Serious extracranial injuries were determined by the presence of any extracranial injury as shown by a radiological examination. Serious extracranial injury is defined by SNC as an abbreviated injury score > 3, corresponding to a quite severe injury [14]. An injury that was significant enough to be visible on a radiological evaluation was deemed as a reasonable substitute.

The NOC and the CCHR were applied in their original forms on the cohort for which they were initially intended. The SNC, the NICE, and the NEXUS II guidelines were applied to the entire cohort. To enable a more thorough comparison, the CCHR and the NOC were also compared using modified inclusion and exclusion criteria, referred to as the adapted CCHR (a-CCHR) and the adapted NOC (a-NOC), in a similar manner as Smits et al. did [18]. Their respective inclusion and exclusion criteria were instead viewed as reasons to order CT (for example an altered level of consciousness in the NOC or the presence of warfarin in the CCHR), making both applicable to the entire cohort. Amnesia, LOC, and disorientation were not needed for inclusion in the a-CCHR or the a-NOC.

The calculations were done using IBM-SPSS version 25 for Windows. The statistical significance was set at *P* = 0.05, and the results were reported with a 95% confidence interval when appropriate. The confidence intervals around the calculated proportions were calculated according to Clopper-Pearson. The normality was assessed with visual inspections of histograms and calculations of skewness and kurtosis. The guidelines’ outcomes were considered independent, nominal variables and analyzed with an χ^2^ test. The performance was assessed using sensitivity, specificity, negative predictive value, and positive predictive value with a 95% confidence interval. An estimated net effect on the CT frequency was calculated for each guideline by subtracting each guideline’s estimated CT frequency from the actual frequency in the cohort.

The reviews were conducted by a single reviewer; 100 random cases were also reviewed by a second reviewer to calculate interrater agreement according to Cohen’s kappa (κ) and the percentage agreement. Agreement in 50% of the cases and the reviewers’ agreement whose difference would not exceed 20% of the value for the whole population were assumed, and the sample size was calculated as comprising a minimum of 93 medical records.

## Results

In total, 1353 patients were eligible for comparative analysis. Of these, 825 (61%) underwent a head CT scan. Seventy (5.2%) cases showed at least one type of intracranial hemorrhage. Three (0.2%) patients required neurosurgical intervention, and four (0.3%) patients died from their head injuries. None of the patients who died due to their head injuries underwent neurosurgery; their high pre-trauma morbidity made them ineligible for surgical intervention. All the patients that either died or required neurosurgical intervention were identified by each of the guidelines when they were applicable. The 89 excluded cases because of previous evaluations consisted of 23 patients who were previously evaluated in another hospital outside our catchment area and 66 patients who were previously evaluated in Helsingborg Hospital. The latter group was reviewed, and no previously missed intracranial hemorrhages were found. Table 1 shows the descriptive statistics and the presence of different risk factors for intracranial hemorrhage.

**Table 1:** Description and presence of risk factors for intracranial hemorrhage

Variable			N	(%)	Missing	(%)
Age	Median	65			0	(0.0)
	Interquartile range	40–81			n/a	n/a
	Min-Max	18–104			n/a	n/a
Gender	Male		747	(53.8)	0	(0.0)
	Female		641	(46.2)	0	(0.0)
Bleeding disorder			9	(0.6)	51	(3.7)
Treatment with anticoagulants			186	(13.4)	47	(3.4)
Treatment with platelet inhibitor			173	(12.5)	45	(3.2)
RLS-85	1		1333	(96.0)	0	(0.0)
	2		49	(3.5)	0	(0.0)
	3		6	(0.4)	0	(0.0)
GCS	15		1331	(95.9)	0	(0.0)
	14		50	(3.6)	0	(0.0)
	9–13		7	(0.5)	0	(0.0)
Loss of consciousness	Certain		272	(19.6)	277	(20.0)
	Suspected		104	(7.5)	n/a	n/a
Amnesia			301	(21.7)	672	(48.4)
Amnesia > 30 min			12	(0.9)	86	(6.2)
Headache			261	(18.8)	811	(58.4)
Over 2 episodes of vomiting			30	(2.2)	692	(49.9)
Dangerous mechanism of injury			57	(4.1)	35	(2.5)
Post-traumatic seizure			14	(1.0)	0	(0.0)
Intoxication			324	(23.3)	758	(54.6)
Abnormal behavior in ED			66	(4.8)	0	(0.0)
Abnormal level of alertness in ED			99	(7.1)	0	(0.0)
Signs of depressed skull fracture			111	(8.0)	1115	(80.3)
Signs of basal skull fracture			18	(1.3)	1225	(88.3)
Scalp hematoma			66	(4.8)	1192	(85.9)
Physical signs of injury above clavicles			1044	(75.2)	188	(13.5)
Neurological deficit			60	(4.3)	919	(66.2)
S-S100B > 0.10 μg/L^a^			229	(58.7)	n/a	n/a
Head CT performed			825	(61)	n/a	n/a
Intracranial hemorrhage^b^		Total	70	(5.1)	n/a	n/a
		Subdural	29	(41.4)	n/a	n/a
		Subarachnoidal	29	(41.4)	n/a	n/a
		Epidural	4	(5.7)	n/a	n/a
		Other	30	(42.9)	n/a	n/a
Skull fracture		Total	18	(1.3)	1	(0.1)
		Basilar	11	(61.1)	n/a	n/a
		Linear, no depression	5	(27.8)	n/a	n/a
		Linear, depression	1	(5.6)	n/a	n/a
		Comminute, no depression	1	(5.6)	n/a	n/a
		Comminute, depression	0	(0.0)	n/a	n/a
Any neurosurgical intervention			3	(0.2)	n/a	n/a
Death due to head injury			4	(0.3)	n/a	n/a

Notes: ^a^Not all patients were sampled. ^b^Some patients had multiple hemorrhages

Abbreviations: *ED* Emergency Department, *CT* Computerized tomography, *RLS-85* Reaction Level Scale 85, *GCS* Glasgow Coma Scale

The NOC was applicable to 256 (18.9%) patients, recommending CT in 249 (97.2%) cases with a sensitivity of 96.5% (82.2–99.9%) and a specificity of 2.6% (1–5.7%) for intracranial injury on CT. This subgroup included 29 (41.4%) intracranial hemorrhages and no cases of death because of TBI or the need for neurosurgical intervention. The CCHR was applicable to 394 (29.1%) patients, recommending CT in 251 (63.7%) cases with a sensitivity of 77.8% (60.9–89.9%) and a specificity of 37.7% (32.7–43%) for intracranial injury on CT. This subgroup included 36 (51.4%) intracranial hemorrhages and no cases of death because of TBI or the need for neurosurgical intervention. The primary outcome (detection of intracranial injuries requiring neurosurgical intervention or resulting in death) could not be assessed for either NOC or CCHR in their original form.

The NICE, the SNC, and the NEXUS II guidelines were applicable to the entire cohort, recommending CT in 595 (44%), 703 (52%), and 891 (65.9%) cases, respectively. All guidelines correctly identified the three patients who required neurosurgical intervention and the four patients who died because of their head injuries. NICE had a sensitivity of 100% (56–100%) and a specificity of 56.3% (54.1–59.3%) for intracranial injury requiring neurosurgical intervention or resulting in death. SNC had a sensitivity of 100% (59–100%) and a specificity of 48.2% (45.6–50.9%) for intracranial injury requiring neurosurgical intervention or resulting in death. NEXUS II had a sensitivity of 100% (56.6–100%) and a specificity of 34.3% (32.1–37.1%) for intracranial injury requiring neurosurgical intervention or resulting in death.

The NICE, the SNC, and the NEXUS II guidelines respectively missed identifying 17, 8, and 10 patients with verified intracranial hemorrhage. Table 2 presents the studied guidelines’ negative predictive value, positive predictive value, sensitivity and specificity in detecting intracranial hemorrhage and their potential effect on the CT rate.

**Table 2:** Each evaluated guideline’s theoretical performance in detecting intracranial hemorrhage and its theoretical effect on CT rate

Guideline	Sensitivity %	Specificity %	NPV %	PPV %	CT rate %	∆ CT rate ^a^ %
a-NOC (95% CI)	97.1 (90.1–99.7)	3.4 (2.4–4.5)	95.6 (84.2–98.9)	5.2 (5–5.4)	96.7 (95.6–97.6)	+ 35.7 (*P* < 0.001)
a-CCHR (95% CI)	87.1 (77–94)	35.7 (33.1–38.9)	98.1 (96.5–99)	6.9 (6.3–7.6)	65.5 (62.9–68)	+ 4.5 (*P* = 0.015)
NEXUS II (95% CI)	85.7 (75.3–92.3)	35.2 (32.6–37.9)	97.8 (96.2–98.8)	6.7 (6.1–7.4)	65.9 (63.3–68.4)	+ 4.9 (*P* = 0.008)
NICE (95% CI)	75.7 (63.1–83.5)	58 (55–60.5)	97.8 (96.7–98.6)	8.9 (7.8–10.2)	44 (41.3–46.7)	- 17 (*P* < 0.001)
SNC (95% CI)	88.6 (78.7–94.9)	50 (47.3–52.8)	98.8 (97.7–99.4)	8.8 (8–9.7)	52 (49.3–54.7)	- 9 (*P* < 0.001)

Abbreviations: *CT* Computerized tomography, *NPV* Negative predictive value, *PPV* Positive predictive value, *a-NOC* Adapted New Orleans Criteria, *a-CCHR* Adapted Canadian CT Head Rule, *NEXUS II* National Emergency X-Radiography Utilization Study II, *NICE* National Institute of Health and Care Excellence, *SNC* Scandinavian Neurotrauma Committee

^a^Analyzed with χ^2^ test

S100B was sampled in a total of 390 (28.8%) cases with 229 (58.7%) above the clinical cut-off (> 0.10 μg/L). S100B was sampled on the correct indication according to the SNC-guideline in 108 (27.7%) cases. Of these, 57 (52.8%) had levels above clinical cut-off and 62 (57.4%) went through CT-scanning. Of the patients where S100B was not measured (*N* = 963), an additional 91 (9.4%) cases were eligible for sampling according to the SNC-guideline. In 18 cases, CT should have been performed based on other criteria without measuring S100B according to SNC but was omitted due to a wrongfully sampled S100B below cut-off. Of the 251 patients who could be classified as having a “mild injury, low risk” according to the SNC guideline, 43 (17.1%) could be discharged without CT based on an S100B level of < 0.10 μg/L.

The interrater agreement varied among the different variables, with κ-values ranging from 0.385 to 1.0. Table 3 presents the full list of κ-values and the corresponding percentage agreements.

**Table 3:** Interrater agreement

Variable	Cohen’s kappa	Percentage agreement
Bleeding disorder ^a^	–	100
Anticoagulation	1.0	100
Thrombocyte inhibitors	0.947	99
Low molecular-weight heparin ^a^	–	99
Mechanism of injury	0.716	81
High energy according to ATLS	0.385	97
Dangerous mechanism	0.662	94
RLS-85	0.655	92
GCS	0.574	90
Loss of consciousness	0.821	93
Amnesia	0.804	92
Headache	0.760	92
Vomiting	0.884	99
Number of vomits	0.542	96
Seizure ^a^	–	97
Intoxication	0.937	98
Abnormal behavior ^a^	–	91
Abnormal consciousness	0.576	90
Signs of depressed skull fracture	0.521	95
Signs of basal skull fracture ^a^	–	99
Scalp hematoma	0.789	98
Signs of trauma above clavicles	0.807	93
Neurological deficit	0.497	93
Presence of intraventricular shunt	1.0	100
S100B measured	0.801	93
S100B level	0.824	93
CT scan of the head	0.978	99
Intracranial hemorrhage	1.0	100
Skull fracture	0.928	99
Other radiology performed	0.768	92
Intubation ^a^	–	99
Neurosurgical intervention	1.0	100
Death	1.0	100

Abbreviations: *ATLS®* Advanced Trauma Life Support, *RLS-85* Reaction Level Scale 85, *GCS* Glasgow Coma Scale

^a^Unable to calculate Cohen’s kappa due to parameter being a constant

## Discussion

We performed a comprehensive review of the medical records and a subsequent estimation of the performance of the commonly used five guidelines when managing patients with mTBI. To the best of our knowledge, this is the first comparison of the stated guidelines in a Scandinavian setting.

Our retrospective study indicates that a reduction in CT scans might be possible through a wide adoption of either the NICE or the SNC guideline when compared with the current management at Helsingborg hospital. Both guidelines would have missed a relatively large amount of intracranial hemorrhages (nine for SNC and 17 for NICE), but none of the missed hemorrhages required neurosurgical intervention or resulted in death. Foks et al. made a similar estimation of the sensitivity of the NICE guideline [28]. The SNC guideline’s estimated sensitivity did not significantly differ from the result of the SNC group’s own external validation conducted in 2015 (88.5% versus 97.2%, *P* = 0.13) [26]. However, it should be noted that patients requiring neurosurgical intervention or who died due to their injury were rare in this cohort. This makes it possible that patients in these categories might have been missed by one or more of the evaluated guidelines in a bigger cohort. To further determine the validity of our findings, a prospective, preferably multicenter trial would be necessary.

The need for identifying intracranial hemorrhages that do not require neurosurgery is unclear. Sherman et al. concluded that the identification of nonsurgical intracranial lesions resulted in only a little positive effect, mainly because long-term sequelae are rare, and there is no proven advantage with early treatment [30]. However, more recent studies point to an increased risk of post-traumatic headache among patients with intracranial hemorrhage, a condition sometimes associated with significant morbidity [31–33]. In a previous article, we reasoned that it could be valuable to identify these patients early and offer them more appropriate work-up, follow-up, and treatment [34].

S100B was sampled in only a minority of the patients, which would be expected in a dataset such as this. However, a substantial amount of these tests was sampled on the wrong indication, with both over- and under-sampling. Ananthaharan et al. observed a similar phenomenon where S100B was wrongfully sampled or not sampled according to the guideline in 15.7% of the cases [15]. The same study also concluded that 20% of the patients in the “mild injury, low risk” group could be discharged based on their low S100B levels, in line with the current findings. In the present study, the majority of the S100B samples were taken when not indicated. The validity of this specific finding is difficult to determine since we had to put up a theoretical model based on retrospective journal entries in order to determine whether a patient was eligible for sampling.

The CCHR and the NOC in their original forms were only applicable to a minority of the patients in this cohort and our primary outcome could not be assessed for either of these in their original form. This might be due to the inappropriate imputation of missing data regarding LOC, amnesia, or the presence of mental deterioration since these are inclusion criteria in the CCHR. It could also be attributed to a change in the mTBI population over time or the difference between the mTBI populations in Sweden and North America. Regardless of the reasons, the CCHR and the NOC are designed to include only a certain subset of patients. This shifts the “subjective” decision from whether a patient should undergo CT to whether the CCHR or the NOC is applicable, which may lead to the wrongful application of these guidelines. In a previous study by our group, the CCHR was wrongfully applied in almost a third of the cases, leading to unnecessary CT scans [35]. Even the SNC guideline, which is supposed to be applicable to all patients with moderate to minimal injuries, is frequently misinterpreted [15, 36]. This shows that guidelines are easily misinterpreted or misunderstood. A more straightforward inclusion can potentially reduce this error. Therefore, we recommend the use of a guideline that can be applied to all patients with minimal to moderate TBI in order to reduce confusion and wrongful application.

Our choice to interpret the omission of CT at the discretion of the treating physician as absence of any intracranial hemorrhage is a limitation, mainly when evaluating our secondary outcome. A possible deterioration from a missed intracranial injury requiring subsequent intervention or leading to death would have been picked up during the data gathering since we screened every patient for readmission due to head injury. It is possible that we might have missed patients with deterioration if they sought medical care outside our health care district, but we deem this unlikely.

This study’s retrospective design and the fact that the presence of different parameters was interpreted by the group member who collected the data certainly reduced our possibility to draw solid conclusions. However, we took several actions to increase both internal and external validity, as suggested by Vassar and Holzmann and Kaji et al. [37, 38]. We constructed a thorough interpretation guide with instructions on how different situations should be coded, had a clearly defined research question before the data collection started and clearly defined inclusion and exclusion criteria, and calculated interrater agreement with both Cohen’s kappa and the percentage agreement. The varying values might partly be due to the differences in how often each parameter was interpreted as positive. For example, treatment with low molecular-weight heparin showed poor Cohen’s kappa but a high percentage agreement, indicating that a small number of different interpretations caused the large reduction. The majority of the parameters showed a good or a very good interrater agreement, and all but one had a percentage agreement over 90. At the same time, some parameters that to a large extent depended on personal interpretation showed only a fair or a moderate agreement.

The medical records had varying levels of quality, as can be interpreted from the varying and often high proportions of missing data. We chose to interpret the missing data as an absence of pathological findings, as stated in the methods section of this paper. Although we discussed the issue and independently arrived at the same conclusion based on experience, this way of imputation is at risk of understating the actual severity of the injury and missing the different criteria that would make the patient eligible for CT. This might partly explain why our expected sensitivity values were substantially lower for each guideline when compared with previous studies evaluating the included guidelines.

To enable analysis and comparison of the different guidelines, assumptions about them, as well as some adjustments, had to be made. The GCS and the RLS-85 are two instruments that share many similarities, rendering them comparable even if they are not identical. They evaluate the level of consciousness based on very similar assessment findings, show a high degree of correlation, and have been found to be transferable to each other [39]. A conversion such as this is done by the SNC in translating its English guideline to Swedish [14, 40].

In our opinion, the choice to extend the treatment with warfarin, including both NOACs and low molecular-weight heparin, is reasonable. NOACs have been associated with lower rates of spontaneous bleeding when compared with warfarin, but they have similar rates of traumatic intracranial hemorrhage [41–44]. The NICE guideline abstained from including NOACs in its algorithm due to insufficient evidence but suggested that the reference standard should be CT with an appropriate follow-up [13]. The CCHR was developed before NOACs became available; therefore, it is impossible to know with certainty whether NOACs would constitute an exclusion criterion.

We abstained from the subclassification of amnesia utilized in the NOC and the CCHR because this finding was not readily available in the medical records. Amnesia lasting for over 30 min, whether antegrade or retrograde, most likely indicates significant trauma and mandates CT.

Significant extracranial injury that would render a patient unfit for S100B is defined as having an abbreviated injury score above 3 in any organ system (e.g., femur fractures or serious abdominal or thoracic injuries). Earlier studies show that the injuries should be quite severe, that is, confirmed fractures or hemorrhagic shock [45, 46]. We can determine the presence of extracranial fractures but cannot grade their severity because the required information is lacking in the primary medical records. With this in mind, the choice to define any fracture or other injury visualized by radiology as significant is the most pragmatic and has the lowest risk of falsely identifying patients as eligible for S100B sampling.

## Conclusion

A broad application of either NICE or SNC guidelines would potentially reduce the number of CT scans for patients suffering from mTBI in a Scandinavian setting, while the other guidelines seemed to increase the CT frequency. The sensitivity for intracranial hemorrhage was lower than in previous studies for all guidelines, but no fatality or need for neurosurgical intervention was missed by any guideline when they were applicable.

## Data Availability

The datasets used and analyzed during the current study are available from the corresponding author on reasonable request.

## Abbreviations

a-CCHR: Adapted Canadian CT Head Rule
a-NOC: Adapted New Orleans Criteria
CCHR: Canadian CT Head Rule
CT: Computerized tomography
GCS: Glasgow Coma Scale
LOC: Loss of consciousness
mTBI: Mild traumatic brain injury
NEXUS II: National Emergency X-Radiography Utilization Study II
NICE: National Institute of Health and Care Excellence
NOAC: New oral anticoagulants
NOC: New Orleans Criteria
RLS-85: Reaction Level Scale 85
S100B: S100 calcium-binding protein B
SNC: Scandinavian Neurotrauma Committee
TBI: Traumatic brain injury
